# Restoration of dystrophin expression using the Sleeping Beauty transposon

**DOI:** 10.1371/currents.RRN1296

**Published:** 2012-02-07

**Authors:** Sofia Muses, Jennifer E Morgan, Dominic J. Wells

**Affiliations:** ^*^Department of Comparative and Biomedical Sciences, The Royal Veterinary College, Royal College Street, London NW1 0TU, U.K. and ^†^The Dubowitz Neuromuscular Centre UCL Institute of Child Health 30 Guilford Street London WC1N 1EH United Kingdom

## Abstract

The Sleeping beauty (SB) system is a non-viral DNA based vector that has been used to stably integrate therapeutic genes into disease models. Here we report the SB system is capable of stably integrating the ΔR4-R23/CTΔ micro-dystrophin gene into a conditionally immortal dystrophin deficient muscle cell-line, H2K SF1, a murine cell model for Duchenne muscular dystrophy. Genetically corrected H2K SF1 cells retained their myogenic properties in vitro. Moreover, upon transplantation ΔR4-R23/CTΔ micro-dystrophin expression was detected within mdx nu/nu mice. Our data suggests the SB system is an effective way of stably integrating therapeutic genes into myogenic cells.

## Introduction

Duchenne muscular dystrophy (DMD) is a lethal muscle wasting disease caused by mutations in the *DMD* gene which prevents the translation of a functional dystrophin protein. Dystrophin deficient muscle is particularly susceptible to contraction induced damage, leading to repetitive rounds of muscle degeneration and regeneration and eventually loss of muscle function. To date no treatment is available that halts or reverses the pathology of DMD.  Gene replacement therapy is a potential treatment option for DMD, however a key factor for successful treatment is the stable integration of the exogenous gene into resident muscle stem cells, thus reducing the need for repeated treatment [1]. Whilst lentiviral vectors are effective in infecting and stably integrating therapeutic genes into cells *ex vivo* and *in vivo*, their high manufacture costs, integration site profile and potential to elicit an immune response hinder their application [2]. This highlights the need for alternative integrating vectors that can overcome these limitations. *Sleeping Beauty* (*SB*) is a Class II DNA transposon which has been used as a DNA vector for stably integrating therapeutic genes into a variety of disease models [3] [4] [5] [6] [7]. *SB* has shown to be a promising tool for non-viral gene therapy as it does not appear to elicit an immune response or have a hazardous integration profile and is therefore deemed safer than retroviral vectors [8] [9] [10]. 

In light of the above, a proof of concept study was conducted to determine if the *SB* system was capable of stably integrating the ΔR4-R23/CTΔ micro-dystrophin gene [11], into a genome of a myogenic cell-line. As a target cell model we used a dystrophin deficient conditionally immortal muscle cell-line, H2K SF1 [12]. The cell-line harbours a thermolabile T-antigen gene [13] which allows continuous myogenic proliferation when incubated at 33^o^C in the presence ofγ-IFN. Upon switching cell culture conditions to 37^o^C and removing γ-IFN the H2K SF1 cells exit mitosis and terminally differentiate.  Although considered, primary myoblasts from the *mdx *mouse [14] [15] were not deemed an appropriate cell model for this study as their limited mitotic capacity *in vitro *would have required repeated isolation of heterogeneous primary myoblasts, which may have increased inter-experimental variability. 

Here we show the *SB* system were capable of stably integrating the ΔR4-R23/CTΔ micro-dystrophin gene into the genome of the H2K SF1 cells. In addition, genetically corrected H2K SF1 cells retained their myogenic potential *in vitro* and *in vivo*, suggesting the non viral *SB* system is a promising non-viral integrating vector for the treatment of neuromuscular diseases. 

## Material and Methods 

### Cell culture

To encourage cell adhesion, myoblasts were cultured on Martigel (0.1mg/mL, B.D. Bioscience, Matrigel diluted in DMEM) coated flasks in proliferating and terminally differentiating conditions. H2K SF1 and the conditionally immortal wild-type cell-line, H2K 2B4 [16], were cultured as previously described [12] [16]. To initiate terminal differentiation H2K SF1 myoblasts were cultured for three days at 37^o^C, 5% CO_2  _in differentiation medium [DMEM, 5% (v/v) Horse Serum, 4mM L-glutamine and 1% penicillin/streptomycin] without γ-IFN, at a seeding density of 5x10^4^ myoblasts per well in a volume of 250µl (LABTEK eight-well chamber slides, Nunc). Myotubes were immunostained as previously described [16] with the following primary antibodies, anti-Pax7 (Developmental Studies Hybridoma Bank,DSHB), anti-MyoD (clone 5.8A, DakoCytomation), anti-myogenin (clone F5D, DSHB), anti-MyHC (clone MF20, DSHB) and T-antigen (EMDA Biosciences). Primary antibodies were detected with an appropriate fluorescent secondary antibody. Nuclei were counterstained with DAPI (Invitrogen).

###  Construction of the pT2/microdys plasmid

The pT2/microdys plasmid consisted of a bicistronic expression cassette in between the terminal repeats of the *SB* transposon [17]. The bicistronic cassette utilised a composite ferritin promoter (MONO) to drive expression of the ΔR4-R23/CTΔ micro-dystrophin and *neo *genes, using an internal ribosome entry site (IRES) to achieve similar gene expression levels. To generate the pT2/microdys plasmid, an intermediate plasmid pT2/MONO-neo-gfp plasmid was constructed.  The *SB* transposon, pT2/BH, and pMONO-neo-gfp (Invivogen) plasmids were digested with the *EcoRV *and *SbfI *restriction enzymes, respectively. Digested fragments were ligated overnight and transformed into chemically competent DH10B *E.coli*. Purified pT2/MONO-neo-gfp cDNA was digested with *AvrII* and *AgeI* and simultaneously the pAAVSpc512 ΔR4-R23/CTΔ micro-dystrophin plasmid [11] was digested with *NotI *and *EcoRI*. To form the pT2/microdys plasmid, pT2/MONO-neo-gfp and ΔR4-R23/CTΔ micro-dystrophin cDNA fragments were ligated overnight prior to transforming into chemically competent DH10B *E.coli*. 

###  pT2/microdys transposition assay 

Using Nucleofection (programme B-032, Lonza, Switzerland), 5x10^5^ H2K SF1 cells were co-transfected with 725ng of pT2/microdys and 50ng of the *SB*100 transposase plasmids [18]. Forty-eight hours post transfection, growth medium was replaced with fresh growth media supplemented with 800µg/mL of G418. Growth media supplemented with 800µg/mL G418 was changed every 2-3 days. G418 resistant clones were counted after 14 days of selection. Integration sites were analysed using the plasmid recovery method [4] [16]. Briefly, genomic DNA (gDNA) was extracted from H2K SF1 G418 resistant colonies, digested with the *SspI* enzyme and self ligated using 3U of ligase (Roche) in a total volume of 500 µl at 22°C overnight. Ligated gDNA was electroporated into 50µl of ElectroMAX DH10B T1 phage competent bacteria (Invitrogen). Transformed bacteria were grown overnight at 30^o^C on LB agar plates containing kanamycin (50µg/mL, Sigma). Kanamycin resistant colonies were counter selected on LB agar plates supplemented with kanamycin and ampicillin (50µg/mL, Sigma). Plasmid DNA was extracted from Kan^resistant ^and Amp^sensitive^  colonies and integrations sites were analysed using primers that hybridised to the left and right terminal repeats of the transposon (Left ITR: 5’GACTTGTGTCATGCACAAAGTAG and Right ITR: 5’CCACTGGGAATGTGATGAAAG [19]). Sequencing reactions were analysed using a nucleotide basic local alignment search tool (nBLAST) against the NCBI mouse genome database.  

### Myoblast transplantation

Experiments were conducted under a Home Office project licence following institutional ethical review (compatible with Directive 86./609/EEC). To increase myoblast engraftment efficiency three week old *mdx nu/nu *mice were anaesthetised with Hypnorm (fentanyl/fluanisone, VetaPharma Ltd) and Hypnovel (midazolam, Roche) and both hindlimbs were irradiated with 18 Gy, as previously described [20]. Three days later the mice were anaesthetised with isoflurane (Abbott Laboratories) and the right irradiated tibialis anterior (TA) muscles were injected with 5x10^5^ genetically modified H2K SF1 cells (total volume of 4µL). The left irradiated tibialis anterior (TA) muscles were injected with 5x10^5^ unmodified H2K SF1 cells. As a control for engraftment efficiency, a mouse was injected with wild-type conditionally immortal cells, H2K 2B4 [16], into both TA muscles. Three weeks post transplantation, ΔR4-R23/CTΔ micro-dystrophin protein expression was assayed immunocytochemically on 10µm transverse muscle cryosections using the polyclonal P7 antibody (1/1000) [21] or the MANEX1011c antibody(1/50)[22]. 

## Results 

To confirm the H2K SF1 clone represented a good myogenic dystrophin deficient model, the cell-line was characterised *in vitro. *A *mdx* ARMS PCR assay [23] confirmed the cells harboured the mutated murine *DMD* gene, which fails to produce a functional dystrophin protein. In addition, detection of the thermolabile T-antigen protein in proliferating myoblasts (33^o^C +γ-IFN) confirmed immortalisation (Figure 1a). The H2K SF1 clone was terminally differentiated and expression of myogenic proteins was assessed (Figure 1b-f). Uniform myotubes and expression of myogenic proteins verified conditional immortalisation did not inhibit the clone’s myogenic capacity. To determine whether the H2K SF1 clone was readily transfectable, 5x10^5 ^H2K SF1 cells were transfected with 2µg of the pMONO-neo-eGFP plasmid using Nucleofection (programme B-032, Lonza, Switzerland). Transient expression of the eGFP gene 48 hours post transfection showed 39% (n=4, SEM ±1.30) of the myoblasts expressing the transgene.

**Figure:**
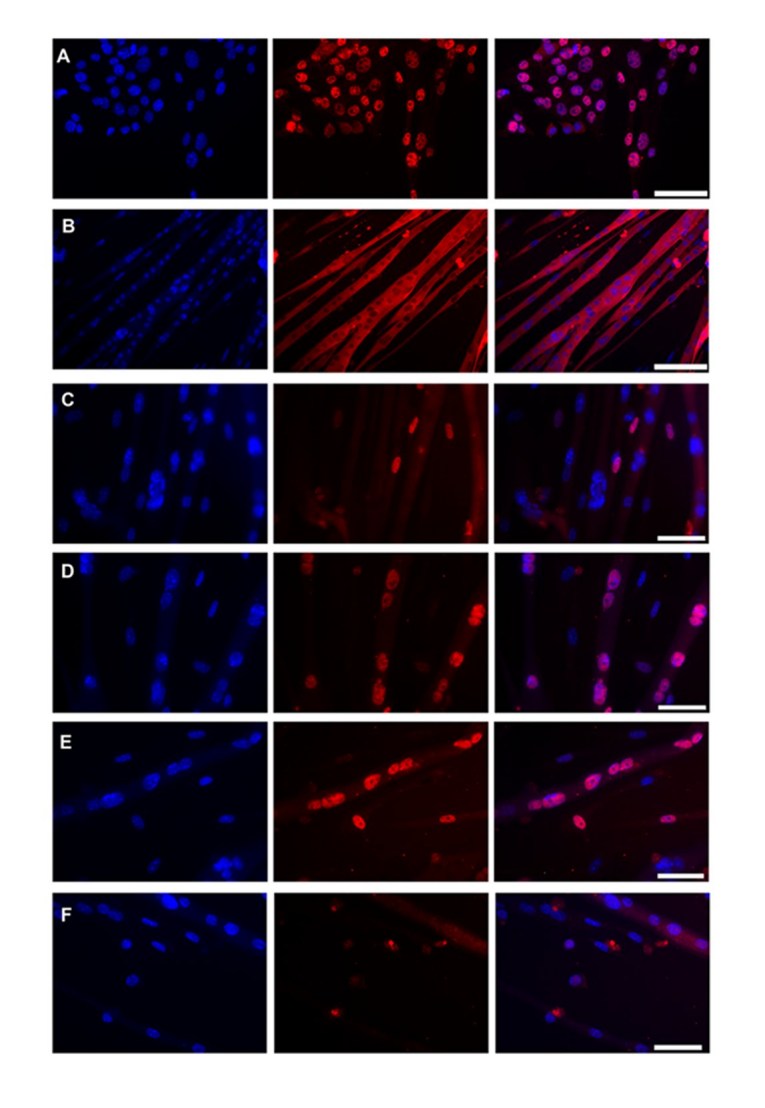
Figure 1 In vitro characterisation of the H2K SF1 cell-line A) Expression of the thermolabile T-antigen protein (red, middle column) in proliferating H2K SF1 cells. Myogenic protein expression in H2K SF1 myotubes,  B) slow& fast myosin, C) Pax7, D) MyoD, E) myogenin and F) T-antigen. Myonuclei were counterstained with DAPI (blue, left column). Right column shows the merged signals. Scale bars: A&B-50 microns, C, D, E&F -25 microns.

To assess if the *SB* system was able to successfully transpose the ΔR4-R23/CTΔ micro-dystrophin gene [11] [24] into the genome of the H2K SF1 cell-line, a new *SB *transposon (pT2/microdys) was constructed. Using Nucleofection, myoblasts were either co-transfected with the pT2/microdys transposon and *SB*100 transposase plasmids or with the pT2/microdys plasmid alone (negative control). Co-transfected H2K SF1 cells had a seven fold increase in the number of G418 resistant colonies compared to the negative control, indicating transposition had occurred (Figure 2a).

**Figure:**
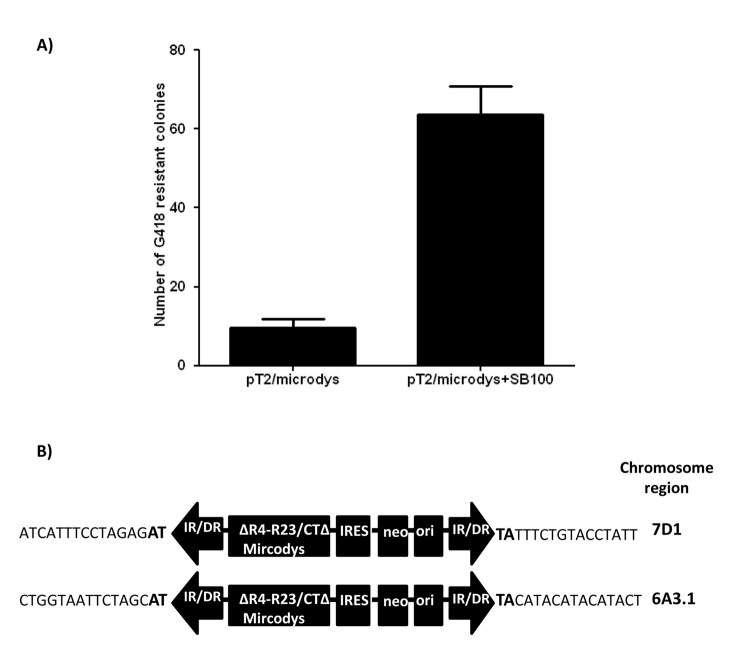
Figure 2 Stable integration of the ΔR4-R23/CTΔ micro-dystrophin gene  A) A histogram showing a significant increase in the number of G418 resistant colonies in H2K SF1 cells co-transfected with pT2/microdys and SB100 transposase plasmids compared to cells transfected only with the pT2/microdys plasmid. Error bars generated from SEM, N value of 4. Mann Whitney test, p=0.0286. B) Molecular evidence of pT2/microdys transposition in H2K SF1 cells. TA dinucleotides (bold) represent the signature integration profile of the SB transposon and the transposon: chromosome junction site.

Transposition of the pT2/microdys transposon was confirmed at a molecular level through the plasmid recovery technique (Figure 2b). When terminally differentiated, the genetically modified H2K SF1 cells formed uniform myotubes and had similar expression levels of the myogenic regulatory proteins as wild type H2K SF1 cells (Figure 3); indicating the stable integration of the ΔR4-R23/CTΔ micro-dystrophin gene did not hinder the cell’s myogenic characteristics* in vitro*.

**Figure:**
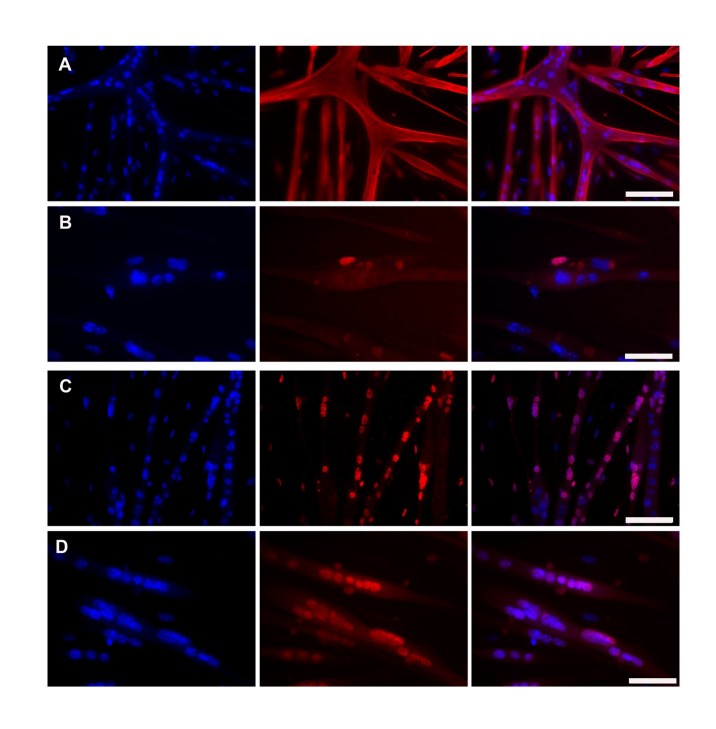
Figure 3 In vitro terminal differentiation of genetically modified H2K SF1 cells Integration of the pT2/microdys gene did not alter the myogenic potential of the H2K SF1 cell-line when terminally differentiated in vitro. Images show the expression of the myogenic related proteins A) desmin (red, middle column), B) Pax7, C) MyoD and D) myogenin. Myonuclei were counterstained with DAPI (blue, left column). Right column shows the merged signals. Scale bars: A & C- 50 microns, B & D-25 microns.

 Subsequently, half a million genetically modified H2K SF1 cells were engrafted into right irradiated TA muscles of *mdx nu/nu* mice. Non-treated H2K SF1 cells were transplanted into the contralateral irradiated TA muscle. As a control for myoblast engraftment efficiency, a wild type conditionally immortal satellite cell derived cell-line, H2K 2B4 [16], was injected into both irradiated TA muscles of an *mdx nu/nu* mouse. Three weeks after engraftment, dystrophin expression was assessed using the P7 and MANEX1110c antibodies [11] [21]. As the engrafted H2K SF1cells were not otherwise genetically marked, we used a double labelling system to distinguish between donor derived ΔR4-R23/CTΔ micro-dystrophin positive myofibres and revertants [25]. Revertants are sporadic dystrophin positive myofibres present in muscles of the *mdx *mouse. These myofibres arise through aberrant splicing of the dystrophin mRNA, removing at least the mutated exon and consequently resulting in an in-frame mRNA and production of a functional dystrophin protein [25]. We could achieve detection of ΔR4-R23/CTΔ micro-dystrophin protein as the P7 antibody binds to the rod domain (exon 57), a region not present within the ΔR4-R23/CTΔ micro-dystrophin protein. In contrast, the MANEX1011C antibody was raised against repeat 1 within the rod domain, which is present in both proteins. Therefore, myofibres detected with both antibodies were likely to be revertants, whilst myofibres detected with only the MANEX1011c antibody were considered to be of donor origin. An average of 71 ΔR4-R23/CTΔ micro-dystrophin positive fibres was detected within two TA muscles engrafted with genetically modified H2K SF1 cells (Figure 4a). No ΔR4-R23/CTΔ micro-dystrophin positive fibres were detected with the P7 antibody (Figure 4b). In addition, besides a few revertants, no dystrophin positive fibres were detected in any of the contralateral TA muscles implanted with untreated H2K SF1cells (data not shown). An average of 88 dystrophin positive myofibres was identified with both antibodies in TA muscles engrafted with the control H2K 2B4 cells (Figure 4c&d). More importantly, engrafted muscles were of normal size and morphology indicating the cells did not form tumours *in vivo.*

**Figure:**
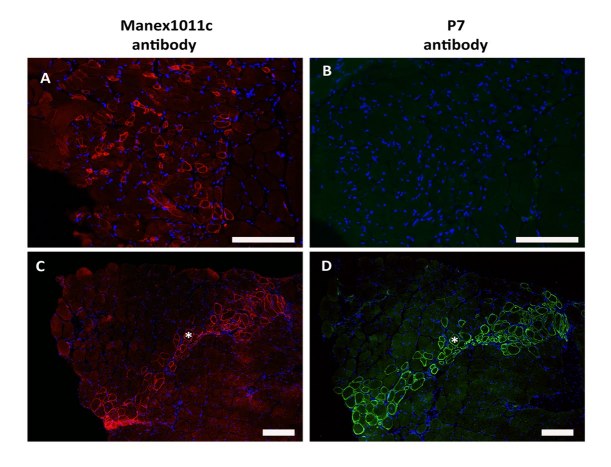
Figure 4 Dystrophin restoration in vivo. A) Images above show ΔR4-R23/CTΔ micro-dystrophin positive myofibres detected with the MANEX1011c antibody within mdx nu/nu TA muscles that had been engrafted with genetically modified H2K SF1 cells. B) No dystrophin positive myofibres can be seen in serial sections when using the P7 antibody. C&D) Dystrophin positive myofibres detected with both antibodies within TA muscles engrafted with wild-type H2K 2B4 cells.  White asterisks indicate same myofibres within serial sections. Myonuclei were counterstained with DAPI (blue). Scale bars-50 microns.

Discussion 

Utilising their inherent ability to integrate into the genome, Class II DNA transposons are fast becoming ideal non-viral integrating tools for gene therapy. The reconstructed transposon, Sleeping Beauty, has been used to integrate reporter and therapeutic genes into the genome of target cells [3] [5] [7] [26] [27]. We have previously shown the *SB* system is capable of stably integrating a reporter plasmid into the conditionally immortal cell-line H2K 2B4 [16]. To further investigate, a proof of concept study was conducted to determine if the transposon based vector was capable of stably integrating the ΔR4-R23/CTΔ micro-dystrophin into a dystrophin deficient cell-line, H2K SF1. A cell-based transposition assay resulted in a seven fold increase in G418 resistant colonies in H2K SF1 cells co-transfected with the *SB* transposon and SB100 transposase compared to H2K SF1 cells transfected with only the *SB* transposon. Stable integration was confirmed at a molecular level using a plasmid recovery method [4] [16]. As we have previously reported the *SB *system integrates randomly within myogenic cells [16], we only looked for *SB* transposition confirmation rather than an integration profile for this study. Once we verified genetically modifying the H2K SF1 with the *SB* system did not alter their ability to terminally differentiate *in vitro*, the cells were engrafted into irradiated TA muscles of *mdx nu/nu* mice. As a control for engraftment efficiency, a wild type conditionally immortal cell-line, H2K 2B4, was injected into irradiated TA muscles. Using a double-labelled antibody system, we confirmed ΔR4-R23/CTΔ micro-dystrophin expression *in vivo*. Whilst this experiment confirmed genetically corrected H2K SF1 cells were able to contribute to muscle regeneration, the engraftment efficiency was lower than expected.  However, this observation may be due to the efficiency of the transplantation as the H2K 2B4 cells also regenerated the *mdx nu/nu* muscles at least three times lower than previously noted [16]. 

 Although myobalsts were used for this proof of concept study, it should be highlighted that these cells are not an appropriate target cell to treat DMD due to their limited migration *in vivo *and hence focal muscle regeneration [28] [29]. In addition, satellite cells and their progeny are not capable of crossing the endothelial walls of blood vessels, thus preventing systemic delivery of the cells [30]. An alternative approach is to use other stem cells that are relatively easy to isolate pure populations, expand *in vitro* and can be delivered systemically for muscle regeneration [31] [32]. 

In this study the ΔR4-R23/CTΔ micro-dystrophin gene was chosen over the mini or full length dystrophin cDNA as the *SB* transposition efficiency decreases with the cargo size [33].

Further development to the *SB *system has resulted in the generation of the *SB* sandwich transposon which has shown to transpose genes up to 10kb [34]. In addition, the modification of another class II DNA transposon, PiggyBac [35], resulting in “*ePiggyBac*”, has been shown to efficiently stably integrate inserts up to 18kb in human embryonic stem cells [36]. Whilst further work is required to test the efficiency of the *SB* sandwich and *ePiggyBac* systems in myogenic cells, these studies highlight the possibility of using transposon based DNA vectors to stably integrate a mini or full length dystrophin gene into the genome of target cells. 

Unlike lentiviral vectors, the *SB* system has been shown to exhibit a random integration profile in a variety of cell types [8] [9] [37], and is therefore deemed a safer alternative. However, further work is required to develop transposons that integrate into safe pre-determined sites within the genome and thus prevent any potential insertional mutagenesis. 

In conclusion, this study is the first to demonstrate that the *SB* system is able to transpose in a murine dystrophin deficient myogenic cell-line. More importantly, the mobile element was able to successfully integrate the therapeutic gene, ΔR4-R23/CTΔ micro-dystrophin into the genome of the H2K SF1 cell-line without hindering the cell’s characteristics *in vitro* or *in vivo. *Although further optimisation is required to improve the transposition efficiency of larger transgenes, the *SB* system has promising qualities as a non-viral DNA vector for the treatment of neuromuscular disorders.    

## Acknowledgments

We thank Dr Perry Hackett (University of Minnesota, USA) and Dr Zoltan Ivics (Max Delbrück Center for Molecular Medicine, Germany) for providing us with the *SB* transposon (pT2) and SB100 transposase plasmids, respectively. We also thank Professor George Dickson (Royal Holloway College, University of London, UK) for providing us with the micro-dystrophin plasmid (pAAV Spc512).  

## Funding information 

This work was supported by a European Union grant (STREP 6). Jennifer Morgan is supported by a Wellcome Trust University award.

## Competing interests

The authors have declared that no competing interests exist.
